# Supplementary material for: Are carbon emissions trading and green financial instruments synergistic? -Comprehensive quantitative research based on content analysis
Source: PLoS One. 2024 Mar 7;19(3):e0298601. doi: 10.1371/journal.pone.0298601 (PMC10919601; doi:10.1371/journal.pone.0298601)
Supplement: S1 Data — (ZIP) [file pone.0298601.s001.zip › Data/地级市Z_F工作报告.zip!/░▓╗╒/┘±╓▌╩╨/└┤╫╘╣1⁄2╓┌║┼í╛┬φ┐╦╩2╛▌═°í┐_2009.docx]

各位代表：
　　现在，我代表市人民政府，向大会报告工作，请予审议，并请列席人员提出意见。
　　一、2008年工作回顾
　　2008年是我国改革开放30周年，也是我市历史上极不平凡的一年。在市委的坚强领导和市人大、市政协的监督支持下，市政府团结带领全市人民，以科学发展观为指导，认真贯彻党的十七大和十七届三中全会精神，全面落实中央、省各项宏观调控政策，同心同德，顽强拼搏，真抓实干，积极应对金融危机的严峻挑战，战胜了年初的冰冻雪灾，有效防控了手足口病疫情，稳妥处置了“问题奶粉”事件，全面完成了市二届人大三次、四次会议确定的年度目标任务。
　　预计全年实现生产总值400亿元，增长12%，人均生产总值7800元；三次产业比由2007年的29.8∶29.1∶41.1调整为2008年的28.8∶30.7∶40.5；全部财政收入24亿元，增长26%，其中地方财政收入12。6亿元，增长27%；固定资产投资115亿元，增长25%；社会消费品零售总额160亿元，增长22%；城镇居民人均可支配收入12700元，农民人均纯收入3500元，分别增长13%和17%。在全球经济增速明显放缓的情况下，我市实现了人均生产总值首超1000美元、二产比重首超一产、进出口总额首超1亿美元三大历史性突破，主要经济指标继续保持两位数以上增幅，人民生活不断改善，各项社会事业全面进步。一年来，我们主要做了以下工作：
　（一）强力实施工业强市战略，工业主导作用进一步增强
　　工业质量和效益同步提升。加快“一都一城一基地”建设，全面提升“125”行动计划，扶优扶强，抓大活小，积极推动企业开拓市场，做大做强。特别是去年四季度以来，我们认真落实国家和省促进经济平稳增长的调控政策，一手抓项目申报，一手抓企业保产促销，获得中央新增项目资金1.58亿元，省中小企业担保基金和贷款风险补偿资金1.26亿元，多数企业保持了产销平稳增长的势头。全市实现工业增加值96.5亿元，增长22%，拉动经济增长5.1个百分点；规模以上工业企业达到288家，比年初增加85家；实现净利润8.3亿元，增长154.4%；工业经济效益综合指数190.1%，比上年提高37.1个百分点。医药制造、农副食品加工、煤炭开采和洗选三大主导产业实现增加值45亿元，占规模工业增加值的72%；50家“125”行动计划承担企业实现销售收入115亿元，新增产值超亿元工业企业6家。
　　项目拉动作用充分显现。坚持项目带动战略，全年实施各类项目889个，完成投资95亿元；其中投资2000万元以上工业项目 68个，完成投资27亿元。全森木业、济人药业饮片二期工程等30个项目竣工投产，板集煤矿、刘店煤矿、雨润宏健休闲食品项目加紧建设，中能安驰微型轿车、金源化工二甲醚项目进行试生产。投资1。8亿元、年产8亿支的雪茄烟新厂建设即将实施，建成后将成为全国最大的手工雪茄烟生产企业。与永煤集团、淮北矿业集团合作取得实质性进展，三星化工“586”、板集电厂等重大项目加快推进。建成投运220千伏、110千伏变电站各1座，在建220千伏、110千伏变电站5座。
　　自主创新和节能减排深入推进。坚持把自主创新和节能减排作为调结构、促增长的战略支点，国家火炬计划亳州中药特色产业基地通过专家组评审；制定国家标准2项、地方标准10项；新创省名牌产品6个，认定市著名商标39件；全面开展与安徽农业大学、辽宁中医药大学等高校的产学研合作，申报省级企业技术中心2家。实施节能技改项目8个，万元GDP综合能耗下降4.3%；利辛县污水处理厂、蒙城县污水处理厂二期工程等一批项目扎实推进，全年削减化学需氧量2200吨、二氧化硫800吨，完成了省下达我市的节能减排约束性指标。
　　（二）大力发展现代农业，三农工作再上新台阶
　　现代农业发展取得新成效。全面落实各项强农惠农政策，粮食总产414.9万吨、小麦亩产452公斤，分别增长15.6%和15.3%，均创历史新高；我市连续3年被省政府评为全省小麦高产攻关活动先进市。畜牧业呈现恢复性增长，全年肉蛋奶总产30万吨，利辛、蒙城两县被列为全国生猪调出大县。新发展市级以上农业产业化龙头企业35家，农民专业合作经济组织83家。新增和改善有效灌溉面积21.9万亩、除涝面积22.7万亩，新建农田林网52万亩，利辛县获省农田水利基本建设“江淮杯”金杯奖。组织新型农民技能培训8.2万人次。经过4年的强力推进，涡河近期治理工程基本完成建设任务。
　　新农村建设实现新发展。完成村镇规划编制433个，整治空心村90个，实施土地开发整理1.6万亩。谯城区古井镇、十八里镇和利辛县城关镇入选省级产业集群镇，全市涌现出利辛王人镇吴营村、谯城区城父镇刘楼村等一批新农村建设新典型，谯城区为民服务全程代理经验在全省推广。
　　（三）积极发展第三产业，现代服务业水平逐步提升
　　文化旅游业加快发展。依托历史文化资源，加强文化旅游景点建设，大力开展宣传促销，推进文化旅游业发展。二夹弦入选国家非物质文化遗产，清音戏、大班会、棒鼓舞等11个项目被列为全省非物质文化遗产名录。市旅游发展总体规划完成初审，陈抟庙建设顺利竣工，曹操纪念馆、华佗中医药文化博物馆、北关历史街区、蒙城庄子祠二期工程扎实推进，古井酒文化博览园被命名为国家4A级景区，花戏楼景区建设完成招标。全年接待国内外游客240.5万人次、实现旅游收入10.8亿元，分别增长 27.5%和20.3%。
　　金融等现代服务业成效显著。坚持把金融业作为促进地方经济发展的重要杠杆，进一步完善金融服务体系。农发行亳州分行、建行亳州市分行挂牌成立，邮政储蓄银行53家分支机构开业，工行升格二级分行获得批准；徽商银行、国元农业保险入驻亳州，华安证券亳州营业部获准设立，利辛众诚小额贷款有限公司获准筹建；市担保公司完成重组，全市已有3家担保公司注册资本金达到1亿元。市建投公司融资平台作用有效发挥，国家开行新增32亿元授信额度，用于我市城市基础设施和重大项目建设。组织开展银企对接活动3次，对接项目实际发放贷款36.5亿元，建行亳州市分行与我市4家单位签订了14亿元的贷款协议。11月末，金融机构各项贷款余额144.8亿元，剔除不良贷款核销因素，较年初实际增加22.8亿元。保险业保费收入12.8亿元，同比增长45.5%，支付赔付金3.3亿元。交通运输、邮电通信、房地产等服务业均保持较快增长势头。
　　（四）切实加强建设与管理，城市面貌明显改观
　　城市建管力度加大。坚持高标准建设、高效能管理，促进城市规模扩大、功能完善、品位提升。中心市区完成市政基础设施和绿化投资约3.2亿元，同比增长1.6倍。魏武广场建成开放，宋汤河城区段改造二期工程已经竣工。汤王大道南段、芍花路西段、人民中路西段道路完善工程、文帝路东段人行道改造工程全面完成。城区供气、供排水等配套基础设施不断完善，新建污水管网7.5公里，改造供水管网279公里，硬化小街道30条。魏武大道和希夷大道南延工程顺利推进，完成投资5088万元。亳州火车站改扩建、泗许高速亳州段项目开工建设。三县县城建设步伐加快，城镇功能不断增强。市区城市管理综合执法体系逐步完善，文明创建工作扎实有效。
　　征地拆迁拆违工作成效明显。制定出台了《亳州市被征地农民养老保障办法》，切实保障被征地拆迁群众利益。谯城区委、区政府克服重重困难，实施征地1060亩，拆迁面积3.58万平方米，全面完成了涡河治理城区段征迁工作和南部新区年度征迁任务，保证了重点项目的顺利实施。出台了《亳州市查处城市规划区内违法用地和违法建设暂行办法》和《亳州市城市规划区内国有闲置土地处置办法》，开展土地“两非”专项整治，拆除违法建筑 3.5万平方米，依法收回土地570亩，非法买卖土地、非法建设势头得到有效遏制。
　　（五）加快推进体制机制创新，改革开放继续深化
　　各项改革纵深推进。以纪念改革开放30周年为契机，全面推进各项改革。国有企业改革步伐加快，九方制药公司正式挂牌出让，古井集团改制总体方案已经职代会审议通过，双轮集团、蒙城制冷机厂等重点国有企业改制扎实推进。农业领域“畜牧兽医体制、农技推广体系、种子管理体制”三项改革基本完成。市属自收自支事业单位改革工作启动，水利、环保、土地、交通、城管、工商等领域的改革迈出新步伐。
　　发展活力日益显现。徽商大会、药博会等重大招商活动取得圆满成功，全年利用市外境内资金100亿元，实际利用外资8300万美元。全民创业扎实推进，新增个体工商户13300户、私营企业 1025家；非公有制经济实现生产总值200亿元，占全市总量的50%。园区经济加快发展，完成固定资产投资13亿元，规模以上企业达79家，中源汽贸城广本4S店、捷众生物制药等项目落户市经济开发区。县区经济实力增强，预计实现生产总值375亿元、全部财政收入16.3亿元，分别占全市的93.5%和67.9%。
　　（六）着力保障和改善民生，和谐社会建设持续推进
　　十八项民生工程和十件实事按时完成。坚持从解决群众切身利益入手，认真组织实施十八项民生工程和十件实事，全年共投入15亿元，其中市、县（区）配套近3.9亿元，惠及全市500多万城乡居民。农村中小学危房改造任务提前超额完成，全市74.1万义务教育阶段学生享受到义务教育保障新政策；18个乡镇卫生院、228个村卫生室、18个社区卫生服务机构全部建成，468万城乡居民参保参合，覆盖城乡的医疗保障体系基本形成；建成农村公路“村村通”工程1194公里；全面完成年内建成30所二级以上标准化敬老院工作目标；新解决22万人的饮水安全问题；建设廉租房516套，4840户住房困难家庭享受廉租住房补贴；18.6万农村低保对象、3.2万名农村五保对象基本生活得到保障。
　　社会事业全面进步。义务教育均衡发展持续推进，高中阶段招生规模继续扩大，职业教育基础能力不断加强，职业技术学院公办体制进一步理顺，亳州师专新校区建设正式启动，电大现代远程教育在校生规模居全省第一。加强重大传染病防控工作，开展乡镇卫生院文明创建活动，公共卫生服务体系不断加强，医疗服务能力和管理水平有了新提高。开工建设乡镇综合文化站5个，建成“杜鹃花工程”省级点5个，群众性文化活动蓬勃开展。深入开展全民健身活动，竞技体育成绩显著。全面推行“村为主”工作机制，强化人口和计划生育管理与服务，低生育水平持续稳定。生态市建设步伐加快。积极支持驻亳解放军和武警部队建设，国防动员、民兵预备役、人民防空和双拥工作取得新成绩。少数民族聚居区扶贫开发工作整体推进。人事、编制、工商、税务、财政、审计、物价、统计、供销、质监、外事侨务、对台、档案、地方志、老龄、慈善、残疾人事业、无线电管理和机关事务管理等各项工作取得新进步。
　　（七）不断强化社会管理，政府自身建设进一步加强
　　妥善应对突发事件，最大限度减少了年初雨雪冰冻灾害带来的损失，手足口病疫情转入常态管理，“问题奶粉”患儿得到及时筛查救治，及早实现了境内被污染水体总砷含量达标。开展道路交通及危险化学品等应急演练，应急管理能力不断提高。“五五”普法活动积极推进，社会主义荣辱观教育不断深入，依法管理宗教事务工作扎实有效。自觉接受人大及其常委会的法律监督、工作监督和政协的民主监督，充分听取民主党派、工商联、无党派人士意见，重视发挥工青妇等人民团体作用，全年共办理人大代表议案建议115件、政协委员提案293件，满意和基本满意率95%以上。深入开展“反腐倡廉制度建设推进年”活动，落实党风廉政建设责任制，从政行为进一步规范。认真落实《政府信息公开条例》，加强市长热线、行风热线和政府网站建设，畅通群众诉求渠道，政府工作透明度不断提高。着力打造公共资源交易平台，行政服务中心职能有效发挥。高度重视信访工作，解决了一大批信访积案。强化农村和社区警务建设，加强社会治安综合治理，深入推进公安“三基”工程建设，严厉打击各类刑事犯罪，稳妥处置涉众型经济犯罪，扎实开展“安全生产隐患治理年”活动，促进了社会和谐稳定。
　　回顾一年来的工作，也必须清醒地认识到我市经济社会发展中还存在一些突出矛盾和问题，主要表现在：项目建设滞后，投资规模较小，发展后劲不足；工业总量不大，骨干企业数目少；农业基础薄弱，产业化水平不高；招商引资、园区建设进展不快，城市设施不完善、辐射带动能力不强；城乡居民收入仍低于全省平均水平；发展环境有待进一步优化。这些问题既是我们工作中存在的突出问题，也是今后工作的着力点和努力方向。
　　各位代表，亳州发展取得的成绩，是省委、省政府和市委正确领导的结果，是市人大、市政协监督支持和社会各界关心帮助的结果，是全市人民奋发努力、团结拼搏的结果。在此，我代表市人民政府，向全市广大干部群众，向各民主党派、工商联、无党派人士和人民团体，向驻亳解放军、武警官兵和政法干警，向所有参与、支持、帮助亳州发展的各界朋友，致以崇高的敬意和衷心的感谢！
　　二、2009年工作的总体要求
　　2009年是顺利实施“十一五”规划最为关键的一年。中央对当前国际国内形势的总体判断是：虽然世界经济环境中存在不稳定、不确定因素，突发性风险前所未有，但经济全球化深入发展的大趋势没有改变；虽然金融危机对我国的影响在加深，但经济发展的基本面没有改变。省委、省政府从我省实际出发，作出了产业向中西部转移的大趋势没有改变，支持我省经济发展的中长期因素没有改变，厚积薄发、加速崛起的趋势没有改变的基本判断。总的来说，形势严峻，“危”中有“机”；挑战前所未有，机遇也前所未有。越是在形势复杂多变的情况下，越要保持清醒头脑；越是在困难情况下，越要坚定知难而进的信心。面对当前形势，我们必须切实增强加快发展的紧迫感和危机感，把困难估计得更充分一些，把举措谋划得更周全一些，做好应对更加复杂和困难局面的各项准备；必须抢抓国家、省扩大内需促进经济增长和省委、省政府加快皖北及沿淮部分市县发展的双重机遇，找准中央和省政策与我市产业优势的契合点，最大限度地争取主动，“危”中寻“机”，化“危”为“机”，为实现三年内“一个赶超、三个翻番、城市面貌大变样”的奋斗目标奠定坚实基础。
　　今年政府工作的总体要求是：深入学习实践科学发展观，全面贯彻落实党的十七大、十七届三中全会和中央、省经济工作会议精神，坚持发展为上、民生为先、稳定为重、少说多做、真抓实干，抢抓发展机遇，创新发展思路，把扩大内需作为保增长的根本途径，把加快发展方式转变和结构调整作为保增长的主攻方向，把深化改革、扩大开放作为保增长的强大动力，把改善民生作为保增长的出发点和落脚点，加快“一都一城一基地”建设，提升“125”行动计划，大力开展“招商引资推进年”和“项目建设推进年”活动，努力促进经济社会又好又快发展。
　　主要预期目标是：全市生产总值增长11%；全部财政收入增长15%；固定资产投资增长30%；社会消费品零售总额增长12%以上；进出口总额增长20%；居民消费价格指数控制在104%左右；城镇居民人均可支配收入增长10%，农民人均纯收入增长7%；新增城镇就业人数2.5万人，城镇登记失业率控制在4%以内；人口自然增长率控制在8.5‰以内；单位生产总值能耗降低4.5%，化学需氧量、二氧化硫排放量分别下降2.3%和0.8%。
　  三、确保经济平稳较快增长
　　积极贯彻和落实进一步扩大内需促进经济增长的政策措施，把扩大内需与增加供给结合起来，把加大投资与促进消费结合起来，妥善应对国际金融危机风险，全力保持经济平稳较快发展。
　　千方百计加大投资力度。抓紧谋划和编报一批符合国家政策导向的大项目，抓紧办理项目土地、环评等审批手续，加强资金调度，确保2009年重点项目储备总投资规模达1000亿元以上，争取列入省“861”行动计划项目30个、总投资200亿元、完成年度投资40亿元以上。加快启动一批重大项目，确保实施总投资2000万元以上的市重点项目超过100个。争取板集煤矿、刘店煤矿、中能安驰汽车、雨润宏健休闲食品等重点项目年内投产，力促豫皖纺织10万锭精梳纱、七里香公司香精香料项目尽快建设。加快推进亳州火车站改造、泗许高速公路亳州段和亳州至阜阳高速公路建设步伐，争取商丘至合肥客运专线尽早开工。积极开展亳州至蚌埠铁路、涡河航道及航运码头、城市立交桥建设项目前期工作。大力推进茨淮新河大型灌区续建配套、中小河流治理、阚疃泵站改造等重点水利工程。加快推进城乡电网建设，新建和扩建220千伏、110千伏变电站7座。
　　积极拓展消费市场。继续实施“万村千乡市场工程”和“新网工程”，组织开展“家电下乡”、“农机下乡”活动，新建和改造一批农家店、农村商品配送中心、农产品批发和农贸市场，努力拓展农村消费市场。落实促进住房消费政策，增加廉租房、经济适用房和普通商品房供给，新开工商品房80万平方米。积极引进苏果等大型流通企业，支持中小商贸企业发展，加快农资、农机、汽车、家具装饰材料等区域性专业市场建设，积极培育汽车、通信、文化、体育、旅游等新的消费热点，不断扩大城乡消费。
　　四、举全市之力实施工业突破
　　坚持把工业作为富民强市的第一推动力，大力实施“工业三年倍增计划”，把做大产业、做强企业和做优品牌结合起来，把工业化和信息化结合起来，努力走出一条符合亳州实际的新型工业化道路。
　　培育壮大优势产业。加快煤炭资源开发，力争年底形成1000万吨生产能力；加快涡阳煤炭综合利用循环经济产业园建设，建成100万吨选煤厂、90万吨煤矸石电厂和5亿块制砖厂，争取板集电厂、三星化工百亿元煤化工项目尽快开工，把煤电、煤化工打造成亳州第一支柱产业。充分利用亳州中医药文化资源和“中华药都”品牌优势，弘扬“道源文化”，建设“养生亳州”，高标准编制现代中药产业发展规划，争取尽早建成国家级现代中药产业基地；引进大型药企来亳投资，加快药业企业创新孵化平台建设，大力发展中成药、中药饮片、药酒、中药日用品和保健品，进一步做大中药及相关产业，形成独具特色的支柱产业。进一步扩大古井集团、双轮集团等企业的市场份额和品牌影响力，把古井镇、高炉镇打造成徽酒名镇，重振亳州白酒产业雄风。加快发展农副产品加工业，做大做强良夫面粉、东升食品、宏健休闲食品、全森木业等一批龙头企业，改造提升金刚石产业，形成市场竞争力较强的优势产业。抓好汽车制造、中重型汽车改装和零部件加工、汽贸城建设等重点项目，把汽车产业打造成为亳州新兴的支柱产业。
　　加快实现规模企业扩量提质。加大扶持力度，实行动态管理，重点培育60家“125”行动计划承担企业，力争新增规模以上工业企业60家。加快技术创新步伐，推进产学研合作，培育省级中药提取工程技术中心1家、高新技术企业5家、高新技术产品8个。大力实施品牌创新战略，新培育注册商标500件、省级以上名牌产品4个。鼓励企业加快技术创新、产品创新和管理创新步伐，降低生产经营成本，提高经济效益和市场竞争力。
　　切实帮助企业排忧解难。针对机械制造、汽车贸易等困难较大的行业，进一步完善帮办帮扶制度，切实解决企业市场销售困难、融资瓶颈加剧、成本压力过大等实际问题。加大对有市场、缺资金企业的政策支持，鼓励企业开足马力，生产出更多适销对路的产品。帮助我市酒业、药业、农产品加工企业加大先进技术和设备引进力度，鼓励优质中药材加工出口，提升产品质量和档次，巩固拓展国内国际市场，努力保持内外贸稳定增长。
　　五、全面加快农村改革发展步伐
　　农业是安天下、稳民心的战略产业，也是我市的优势产业。越是在经济困难的时候，农业的战略地位越显重要。我们必须继续弘扬大包干精神，强化农业基础地位，大力发展现代农业，努力实现农村改革发展新突破。
　　大力发展高效规模农业。继续实施小麦高产攻关和玉米振兴计划，加快以农田水利为重点的农业基础建设，提高粮食综合生产能力，力争全年粮食总产达90亿斤。大力发展规模养殖，完善基层动物疫病防控体系，年内新增标准化养殖场（小区）60个。突出发展中药材、蔬菜、林木等优质高效作物，积极培育发展有特色、无公害的品牌产品。尤其是要大力推广中药材标准化种植，以中药龙头企业发展带动农业结构调整，从源头上保证药品质量。抓住农业生产基地建设、农产品市场体系建设和农产品加工三个环节，突出龙头企业、合作组织和农户三个利益主体，引导龙头企业建基地、连农户，拉长农副产品加工链条，形成一批具有一定规模、带动能力强的农业产业化龙头企业，年内订单农业面积达到300万亩、农副产品加工产值达122亿元。以阳光工程和新型农民培训为重点，着力加强农民职业技能培训，年内培训农民8.5万人次、新增劳务输出5.6万人。
　　推进农村制度建设和机制创新。建立市县乡村四级土地流转服务平台，在依法自愿有偿的基础上，引导和鼓励广大农户流转土地承包经营权，力争年内规模经营面积达10万亩。加快组建多种形式的农民专业合作组织，全年新增90家以上。深化为民服务全程代理制，全面落实农业投入保障、农产品价格保护和农业补贴等制度，深入推进农村综合改革，积极创新农村基层服务机制。加快村镇规划编制工作，年内完成447个村、19个镇的规划编制。统筹安排城乡基础设施建设，逐步建立城乡统一的公共服务制度。
　　不断改善农村生产生活条件。继续实施“百村十镇”示范工程，以饮安全水、走平坦路、建沼气池为重点，加强农村环境建设，改造升级200公里县乡公路，新建改造800公里“村村通”公路，解决25万农民的饮水安全问题，新增沼气池9070口，不断改善农村卫生条件和人居环境。高度重视和关心村组干部工作生活，不断提高他们的政治经济待遇。
　　六、增强城镇综合承载和辐射带动能力
　　以建设宜居城市为目标，按照加快建设新城区、完善提高建成区、保护改造历史街区、稳步推进开发区的思路，加强基础设施建设，完善城市功能，提升城市品位。
　　加快城镇建设步伐。完成新一轮土地利用总体规划编制，加快城市建设总体规划修编，引导各类资金投入城市基础设施和公益事业建设，全年拟完成中心城区建设投资17亿元，实施项目35个，力争尽快把中心城区建设用地规模扩大到130平方公里。年内重点抓好城区10项建设工程：（1）投资6.75亿元，强力推进市南部新区道路建设工程，完成魏武大道、希夷大道向南延伸及南部新区工业集中区主干道路建设，启动G105与S307道路连接线、汤王大道南段、龙山路、黄河路建设工程；（2）投资3。1亿元，完成亳州火车站改扩建工程；（3）投资2034万元，完成新华路等城区8条主干道人行道改造工程；（4）投资1855万元，完成市北关历史街区基础设施建设工程；（5）投资2.66亿元，开工建设市人民医院新院区一期工程；（6）投资1.19亿元，开工建设亳州师专新校区一期工程；（7）投资5000万元，启动市区涡河景观带建设一期工程；（8）投资1300万元，启动市博物馆建设工程，完成曹操纪念馆改造及陈列工程；（9）投资5800万元，继续实施市区自来水管网改造工程，开工建设涡北水厂；（10）投资2.9亿元，完成市经济开发区、南部新区和汤王大道3个拆迁安置小区建设。同时，进一步加大县城和小城镇建设力度，加快重点中心镇基础设施和工业集中区建设，积极发展特色镇，促进产业集聚发展。
　　推行城市精细化管理。以创建全省文明城市和省级园林城市为抓手，集中整治市容市貌，着力解决车辆乱停乱放、门面占道经营等突出问题。规范城区建房秩序，依法实施征地拆迁，继续严厉查处城市规划区内非法用地、非法建设行为。坚持公交优先发展，合理布局公交线路，加强交通秩序整治，切实解决城区“乘车难”、“行车难”、“停车难”问题。加强社区建设，充分发挥社区在文明创建、治安防控等方面的作用。加强城区绿化工作，创造整洁、优美、有序的城市环境。
　　全面提升开发区竞争力。加快市经济开发区道路建设步伐，全力推动电信大楼、捷众生物制药等重点项目建设，争取中源汽贸城广本4S店尽早投入运营；继续完善县区工业园基础设施，加快标准化厂房建设和工业项目组装，促进生产要素聚集，尽快形成煤电煤化工、中药饮片提取、汽车加工贸易、机械加工制造、纱门丝网加工等一批特色产业园和工业集中区，确保园区完成固定资产投资30亿元、工业增加值增长35%以上。
　　加快发展文化旅游业。发挥文化名城、名人故里、中华药都、白酒之乡、养生亳州五大品牌优势，完善旅游发展总体规划和专项规划，做好药、酒、道家文化等结合文章，形成独具亳州特色的文化旅游产业。引进市外有实力的大企业投资，加快组建亳州文化旅游传媒投资公司，整合文化旅游资源，改造提升现有景区景点，精心编排旅游线路，推行市内旅游门票“一票制”，实施“优秀旅游村镇”创建工程，加强旅游促销推介，打造新皖北旅游板块中心城市，确保旅游业总收入增长20%以上。
　　七、进一步深化改革开放
　　认真贯彻胡锦涛总书记在纪念党的十一届三中全会召开30周年大会上的讲话精神，加大改革攻坚力度，实施全方位开放战略，坚定信心、砥砺勇气，激发活力、加快发展。
　　深入推进重点领域和关键环节改革。加快以古井集团、双轮集团、康达制动器公司为重点的国企改革重组步伐，努力解决国有企业改革的遗留问题，不断提高企业市场竞争力。进一步完善金融体系，争取徽商银行在三县设立分支机构，引进股份制商业银行，促进谯城区农村信用联社组建为农村合作银行，积极发展村镇银行、小额贷款公司和农村互助资金组织。支持金融机构开展银团贷款、企业联保、股权质押、应收帐款质押等多种业务，加大信贷投放，优化信贷结构，确保年内新增贷款60亿元以上。继续引进证券、保险机构入驻亳州，充分发挥他们支持地方经济发展的积极作用。用足用活省中小企业担保转移支付资金，壮大担保公司资本金，放大资金使用效益，提高担保能力。进一步完善政府投融资平台，支持市建投公司壮大实力。推进银企合作，优化金融生态环境，打击恶意逃废债行为，保障金融资金安全。 加快事业单位改革，基本完成市属自收自支单位改制任务。
　　全力开创招商引资新局面。创新招商引资方式，发挥企业主体作用，突出招老乡、以商招商、小分队招商和专题招商，重点抓好第四届中博会、国际（亳州）中医药博览会等重大招商活动。围绕优势产业，精心策划推出一批工业项目，加大文化旅游、商贸流通和基础设施建设招商力度，促进招商引资向三次产业全面拓展。瞄准长三角、珠三角和山东、河南等重点地区，每季度组织一次推介会，实行上门招商，提高招商引资实效。完善优惠政策，强化目标管理，严格绩效考核，推行招商引资“一票认可制”和“代办单位服务终身负责制”，确保全市引进并开工建设固定资产投资5000万元以上项目60个，利用市外资金150亿元以上。
　　不断提高对内对外开放水平。加大扶持力度，完善激励措施，提供优质服务，大力推进全民创业，全力促进非公有制经济发展，确保年内新建创业园区和创业辅导基地10个，新增个体工商户15000家、私营企业1200家。以个体私营和外向型经济为主体，进一步发展壮大县区经济，促进各县区优势互补、协调发展。实施“走出去”战略，积极参与泛长三角分工合作，主动承接产业转移，大力发展外向型经济，力争实现进出口总额1.2亿美元以上。
　　八、全面加强和谐亳州建设
　　改善民生既是保增长的需要，也是保增长、促和谐的出发点和落脚点。要突出抓好就业和社会保障工作，统筹发展各项社会事业，办好关系人民群众切身利益的实事，让人民群众实实在在享受更多实惠。
　　全力实施民生工程。今年，省委、省政府将继续实施民生工程，除已经完成的农村中小学D级危房改造任务外，在去年的基础上新增11项、扩大到28项，财政投入总规模达到220亿元左右。预计我市投入规模将超过16亿元，惠及500多万城乡居民。我们将进一步提高农村低保、五保供养标准，完善城乡义务经费保障机制，继续推进新型农村合作医疗和城镇居民医疗保险制度，逐步提高城乡医疗救助水平，抓好城乡卫生服务体系、农村人口饮水安全工程和农村公路建设，加大廉租房建设力度。主动加强与上级部门衔接，及时足额落实地方配套资金，突出抓好乡镇文化站、农民体育健身、政策性农业保险和提高妇女儿童健康水平等新增的11项民生工程，确保不折不扣地完成年度任务。
　　完善就业和社会保障体系。坚持以创业促就业，实施更加积极的就业政策，加快发展就业容量大的劳动密集型产业、中小企业和服务业。完善就业公共服务平台，加强就业、创业培训，年内免费培训下岗失业人员6900人、农民工2.22万人。高度重视农民工就业问题，支持外出务工人员返乡创业，变“返乡潮”为“创业潮”。加强就业指导和就业援助，妥善处理劳动争议。重点做好下岗失业人员再就业和零就业家庭、就业困难人员、被征地农民、高校毕业生、退伍军人就业与安置工作。加大扶贫开发力度，逐步提高城乡居民特别是困难群体收入水平。加强社会保险费筹集，扩大社会保险覆盖面，全市养老、失业、医疗保险参保人数分别达到8万人、14.5万人和18万人。扎实推进农村敬老院建设，确保全市五保对象集中供养率达到25%以上。
　　统筹发展各项社会事业。推进义务教育学校标准化建设，扩充城区公办义务教育资源，继续改善城乡义务教育办学条件，新建或改扩建农村中小学寄宿制学校10个、城区中小学6个；提高普通高中整体教育质量，积极发展民办教育，加速推进亳州师专新校区建设及改造升格工作，加快亳州职业技术学院发展步伐，积极筹建亳州药业职业技术学院，充分发挥教育服务地方经济发展的作用；加强教师队伍建设，不断提高他们的业务水平和道德素质。加快实施华佗中医院扩建工程，扎实推进城乡卫生服务体系建设，深入开展乡镇卫生院文明创建和标准化建设活动，继续做好重大传染病救治救助工作，不断提高新农合工作水平，逐步完善覆盖城乡的公共卫生服务体系。积极筹建文广大厦，加快推进数字电视整体平移工作，尽快实现亳州电视台一套节目无线全覆盖。重视文物和非物质文化遗产保护，大力开展多种形式的文化下乡活动。加强城乡基层体育设施建设，开展全民健身活动，提高竞技体育水平。进一步加强人口和计划生育工作，加大节育奖励力度，稳定低生育水平，努力降低出生人口性别比。大力推进环保治理工程，强力推进生态市建设。高度重视统计工作，扎实做好第二次全国经济普查。加强价格监管，保持物价总水平基本稳定。大力发展妇女儿童、老龄、残疾人和慈善事业。进一步做好人事、编制、工商、税务、审计、物价、气象、人防、农机、外事、侨务、对台、档案、地方志、防震减灾、无线电管理、机关事务管理等各项工作。
　　切实加强社会管理。深入推进“平安亳州”建设，切实加强社会治安综合治理，夯实基层基础工作，严厉打击各种刑事犯罪，切实增强人民群众的安全感。完善信访工作长效机制，引导群众依法有序合理反映诉求，妥善解决群众反映强烈的突出问题。加强安全生产监管，严格安全生产责任制和责任追究制，继续开展安全生产隐患大检查大整治活动，确保安全生产形势持续稳定好转。加强应急管理，提高预防和处置突发事件的能力。继续开展产品质量和食品安全专项整治，整顿规范药品药材市场秩序，保障人民群众饮食和用药安全。深入开展“五五”普法，积极推行村务企务公开和民主管理，保障群众依法行使权利。广泛开展群众性精神文明创建活动，加强诚信亳州建设，不断提高全社会道德水准和文明水平。大力开展国防教育，推进“双拥共建”，巩固发展军政、军民团结。开展民族团结进步活动，依法加强宗教事务管理。
　　九、着力提高领导科学发展的能力
　　完成今年的各项任务，我们必须以深入开展学习实践科学发展观活动为契机，进一步解放思想，提振精神状态，凝聚发展合力，抢抓发展机遇，优化发展环境，破解发展难题，切实把发展的思路和目标转化为全市人民的共同行动。
　　强化发展意识。发展是解决一切问题的“总钥匙”，是各级领导干部的第一职责。不发展最不科学，抓发展必须科学。不论环境如何变化，我们都必须咬定发展不放松，坚持发展不动摇，加快发展不停步。必须牢固树立锐意进取、敢为人先的发展意识，切实增强不进则退、慢进亦退的忧患意识，始终保持敢于闯、不服输的胆识和气魄，始终保持开拓创新、迎难而上的干劲和斗志，始终保持敢于负责、一抓到底的作风和精神，提升精气神，调动积极性，进一步坚定战胜困难的信心和决心，形成奋发有为、昂扬向上的良好氛围，牢牢把握工作的主动权。
　　提升服务能力。应对危机、加快发展不仅需要信心，更需要办法。各级干部必须学习新知识，探索新思路，增强新本领，解决新问题。坚持以人为本，顺应民意，发扬民主，改善民生，让支持改革、拥护发展的广大群众共享改革发展成果。牢固树立“抓服务就是抓环境、抓环境就是抓发展”的理念，继续深化机关效能建设，完善效能考评办法，加强对中间环节、中层干部的管理，组织开展企业评机关、评中层干部活动，着力解决工作中存在的中间梗阻、执行不力、服务不优、效率不高等突出问题。要管好政府有形的手，用好市场无形的手，努力营造鼓励探索、支持创新、宽容失误、尊重纳税人和企业家的良好环境，进一步调动各方面加快发展的积极性和创造性。
　　狠抓工作落实。各级各部门特别是领导干部要坚持干字当头、以实为先，把精力和心思凝聚到抓大事、办实事、抓落实、求实效上，做到责任不推卸、问题不推诿、矛盾不回避，千方百计把各项工作落到实处。进一步严格目标管理责任制，健全考核评价体系，强化对招商引资、固定资产投资、征地拆迁和民生工程等重点工作的日常考核调度，确保抓落实工作长期化、制度化。
　　依法廉洁从政。自觉接受人大及其常委会的法律监督、工作监督和政协的民主监督，认真办理人大代表议案、建议和政协委员提案，主动听取各民主党派、工商联和社会各界人士的意见。大力实施政府信息公开，清理行政许可项目，全面推行并联审批和为民、为企全程代理服务，完善重大事项集体决策、专家咨询、社会公示与听证等制度，提高政府公信力和群众满意度。全面落实党风廉政建设责任制，巩固反腐倡廉制度建设推进年活动成果，坚决打击权钱交易、商业贿赂等腐败行为，坚决纠正损害群众利益的不正之风。大力弘扬节俭创业精神，切实降低行政成本，努力建设节约型政府，把有限的资金和资源用在加快发展、改善民生上，全面树立为民、务实、清廉的政府形象。
　　各位代表，历史铺垫坚实的基础，人民寄予殷切的期望。亳州经济社会发展正处于重要的战略机遇期，我们恰逢其时，任务光荣艰巨。让我们在中共亳州市委的领导下，紧紧团结和依靠全市人民，坚定信心，迎难而上，锐意进取，扎实工作，全面做好今年经济社会发展工作，以优异的成绩迎接新中国成立60周年！
　　附：名词术语解释
　　1、三年内“一个赶超、三个翻番、城市面貌大变样”的奋斗目标：即通过加快工业化、城镇化和农业产业化发展步伐，三年内全市经济发展速度赶上或超过全省平均水平，工业增加值、固定资产投资和财政收入在2007年基础上翻一番，实现城市面貌大变样，不断开创亳州加快发展的新局面。
　　2、“工业三年倍增计划”：即通过牢固树立“工业强市”理念，大力实施“125”行动计划，加大招商引资和项目建设力度，加快开发区和工业园区建设，确保到2010年全市工业经济总量比2007年翻一番，工业增加值达150亿元以上，年均增长24.3%以上。
